# Evaluation of RNA*later* as a Field-Compatible Preservation Method for Metaproteomic Analyses of Bacterium-Animal Symbioses

**DOI:** 10.1128/Spectrum.01429-21

**Published:** 2021-10-27

**Authors:** Marlene Jensen, Juliane Wippler, Manuel Kleiner

**Affiliations:** a Department of Plant and Microbial Biology, North Carolina State University, Raleigh, North Carolina, USA; b Symbiosis Department, Max Planck Institute for Marine Microbiology, Bremen, Germany; University of Minnesota

**Keywords:** mass spectrometry, 1D-LC-MS/MS, microbial communities, preservation methods, microbiome, field sampling, environmental microbiology

## Abstract

Field studies are central to environmental microbiology and microbial ecology, because they enable studies of natural microbial communities. Metaproteomics, the study of protein abundances in microbial communities, allows investigators to study these communities “*in situ*,” which requires protein preservation directly in the field because protein abundance patterns can change rapidly after sampling. Ideally, a protein preservative for field deployment works rapidly and preserves the whole proteome, is stable in long-term storage, is nonhazardous and easy to transport, and is available at low cost. Although these requirements might be met by several protein preservatives, an assessment of their suitability under field conditions when targeted for metaproteomic analyses is currently lacking. Here, we compared the protein preservation performance of flash freezing and the preservation solution RNA*later* using the marine gutless oligochaete Olavius algarvensis and its symbiotic microbes as a test case. In addition, we evaluated long-term RNA*later* storage after 1 day, 1 week, and 4 weeks at room temperature (22°C to 23°C). We evaluated protein preservation using one-dimensional liquid chromatography-tandem mass spectrometry. We found that RNA*later* and flash freezing preserved proteins equally well in terms of total numbers of identified proteins and relative abundances of individual proteins, and none of the test time points was altered, compared to time zero. Moreover, we did not find biases against specific taxonomic groups or proteins with particular biochemical properties. Based on our metaproteomic data and the logistical requirements for field deployment, we recommend RNA*later* for protein preservation of field-collected samples targeted for metaproteomic analyses.

**IMPORTANCE** Metaproteomics, the large-scale identification and quantification of proteins from microbial communities, provide direct insights into the phenotypes of microorganisms on the molecular level. To ensure the integrity of the metaproteomic data, samples need to be preserved immediately after sampling to avoid changes in protein abundance patterns. In laboratory setups, samples for proteomic analyses are most commonly preserved by flash freezing; however, liquid nitrogen or dry ice is often unavailable at remote field locations, due to their hazardous nature and transport restrictions. Our study shows that RNA*later* can serve as a low-hazard, easy-to-transport alternative to flash freezing for field preservation of samples for metaproteomic analyses. We show that RNA*later* preserves the metaproteome equally well, compared to flash freezing, and protein abundance patterns remain stable during long-term storage for at least 4 weeks at room temperature.

## INTRODUCTION

Field studies are central to environmental microbiology and microbial ecology because they allow for the *in situ* study of microbial communities and their interactions with biotic and abiotic environments. This includes the study of symbiotic interactions between microorganisms and animal or plant hosts. Many of the approaches used to study these symbioses “*in situ*” actually require that samples be preserved in the field for later analysis in the laboratory. This includes, for example, cell counting of specific taxa using fluorescence *in situ* hybridization (FISH) methods (1–3) and various sequencing-based approaches for the characterization of taxonomic community structures and functional potential, such as 16S rRNA gene sequencing (4–6) and shotgun metagenomics (7–11). Sample preservation methods for most of these approaches have been established over the past few decades (12–14). For some approaches, however, including metaproteomics, field preservation methods have not been extensively investigated.

Metaproteomics is an umbrella term that encompasses approaches for the large-scale identification and quantification of expressed proteins in a microbial community (15, 16). Metaproteomics can be used not only to determine the metabolism and physiology of community members but also to estimate community member abundances, interactions, and carbon sources (16–18). Over the past decade, metaproteomics has led to many significant discoveries, including novel insights into microbial biofilm communities collected from acid mine drainages (19, 20), marine symbioses (11, 21, 22), soil communities (23, 24), and the human gut microbiota (25–27).

One critical consideration for field-based metaproteomic studies is the preservation of *in situ* gene expression patterns by stopping biological activity in the samples. In contrast to DNA-based approaches, such as 16S rRNA gene sequencing and shotgun metagenomics, for which outcomes change only if cell numbers in the sample change considerably through cell growth and division, protein abundances in cells can change more quickly in response to changing environmental conditions; therefore, fast preservation in the field is essential for an accurate snapshot of community activity. The time windows in which protein abundance changes are detectable varies greatly between species. For example, for the fast-growing Escherichia coli, protein abundances have been shown to significantly change within 10 to 30 min after exposure to environmental stress (28), while changes in slow-growing ammonia-oxidizing bacteria take many hours to days to occur (29). Therefore, sample preservation for metaproteomics should ideally happen within 10 to 30 min after removal of a specimen from its environment, depending on the response time of the species in question. While this can easily be achieved in the laboratory simply by flash freezing the sample, it can be challenging when collecting samples in the field. At remote field sites, low-temperature freezers are often unavailable and liquid nitrogen or dry ice for flash freezing is not an option due to restrictions on transport to the field site or boiling off/sublimation during extended field stays. Therefore, a field-compatible method for metaproteomic sample preservation is needed. Such a method should (i) immediately stop biological activity and thus prevent changes in gene expression and protein degradation, (ii) preserve the whole metaproteome without bias against specific protein types or taxonomic groups, (iii) work for extended storage at room temperature, (iv) work for a wide range of sample types and species, (v) be nonhazardous and easy to transport, and (vi) be available at low cost.

Various studies have evaluated field-compatible preservation methods for nucleic acids in a wide variety of sample types (14, 30, 31). In contrast, only limited work has been done on field preservation of proteins and, to our knowledge, only for a single cultured bacterial species and fecal samples (32, 33). Saito et al. (32) examined the performance of SDS extraction buffer, ethanol, trichloroacetic acid (TCA), B-PER reagent, and RNA*later* storage solution to preserve cultures of the marine cyanobacterium *Synechococcus*, using flash-frozen samples as a control. The test samples were stored at room temperature for 4 weeks. The authors found that all samples yielded lower protein concentrations, compared to their flash-frozen control. Despite this, Saito et al. (32) found that with RNA*later* the number of identified proteins and relative protein abundances were highly similar to those of flash-frozen controls, while the remaining preservatives showed significantly lower protein identification numbers.

Although studies of different preservation methods and their effects on protein levels remain limited, the work of Saito et al. (32) suggests that RNA*later* is a promising candidate for a field-compatible preservative. In fact, RNA*later* preservation has been used for (meta)proteomic studies of several bacterium-animal symbioses, although without proper validation; therefore, potential impacts on the metaproteomes remain unknown (34–37).

The objective of our study was to identify and to validate a field-compatible preservation method for metaproteomic analyses of bacterium-animal symbioses. We chose to evaluate the protein preservation performances of flash freezing and RNA*later*. To accurately simulate storage of field samples, which are often kept for days to weeks at room temperature due to the lack of refrigeration, we also conducted a time series with RNA*later* at room temperature for up to 4 weeks. We used the Olavius algarvensis symbiosis as a test case. O. algarvensis is a gutless marine worm that harbors two aerobic sulfur-oxidizing gammaproteobacteria (“*Candidatus* Thiosymbion algarvensis” [formerly γ1] and γ3 symbiont), two anaerobic sulfate-reducing deltaproteobacteria (δ1 and δ4 symbionts), and a spirochaete (38–41). We chose to use the O. algarvensis symbiosis as a model because it has been extensively studied with -omic techniques such as metagenomics, metatranscriptomics, and metaproteomics (21, 40, 41). This provides the benefit of a well-validated test system for which a custom protein sequence database is available for protein identification. Moreover, this symbiosis is highly specific, with the animal host always being associated with a set of the same bacterial symbionts, which allowed us to robustly evaluate the preservation performance for both eukaryotic and prokaryotic proteins. Due to its small size (1.5 to 2.5 cm long and 0.1 to 0.13 mm thick) (42), O. algarvensis also allowed us to account for samples with little biomass, which is another common limitation when working with field-collected samples. Overall, these data enabled us to provide recommendations to researchers in various fields of biology who work with field-collected samples targeted for metaproteomic analyses.

## RESULTS

We compared flash freezing and RNA*later* preservation to determine whether RNA*later* is a suitable method for preservation of field-collected samples targeted for metaproteomic analyses. We used the marine gutless oligochaete O. algarvensis and its bacterial endosymbionts as our test system. To simulate field conditions, we also conducted a time series to assess how metaproteomes were affected by storage of samples in RNA*later* for up to 4 weeks at room temperature.

### Similar numbers of proteins identified for both preservation methods and all RNA*later* storage time points.

**FIG 1:**
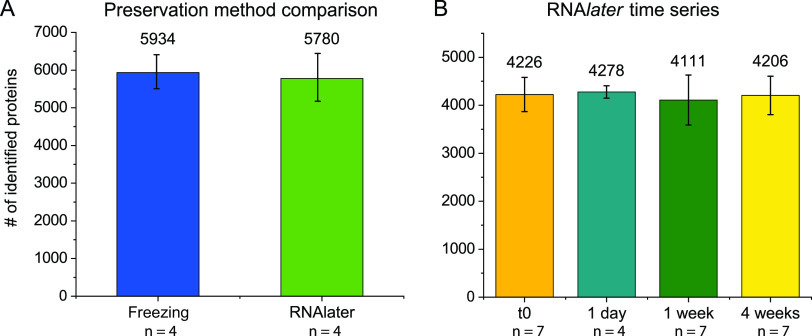
Numbers of identified proteins did not differ between preservation methods and time points. Identified proteins were filtered for an FDR of 5% prior to counting. Error bars indicate the SD of the mean. (A) Average numbers of identified proteins from flash-frozen and RNA*later*-preserved samples. (B) Average numbers of identified proteins in samples from the four RNA*later* storage time points. Samples were frozen immediately after incubation in RNA*later* at 4°C overnight for the time zero time point and after 1 extra day, 1 week, and 4 weeks of RNA*later* incubation at room temperature. It is noteworthy that the numbers of total identified proteins vary between panels A and B due to small but relevant differences in the protein extraction protocols. These differences led to more efficient extraction of abundant muscle proteins of the host in samples of the RNA*later* time series. The large overabundance of peptides from these muscle proteins increased the detection limit for other proteins with, lower abundance.

We identified similar numbers of proteins for both flash-frozen and RNA*later*-preserved samples. On average, we identified 5,934 proteins in flash-frozen samples and 5,780 proteins in RNA*later*-preserved samples (Fig. 1A). The average numbers of identified proteins for flash-frozen samples and RNA*later*-preserved samples were not significantly different (Student's *t* test, *P* > 0.05). This finding suggests that neither of the tested methods outperforms the other in terms of total number of identified proteins.

The numbers of identified proteins were stable across the four tested storage time points. On average, we identified between 4,111 and 4,278 proteins per time point (Fig. 1B). None of the total protein numbers was significantly different from that at time zero, the starting point of the RNA*later* incubation (Student's *t* test, *P* > 0.05). While the manufacturer recommends that samples should be stored at 4°C if storage exceeds 1 week, our results suggest that proteins are well preserved for at least 4 weeks at room temperature.

### No systematic bias in protein identification and quantification between preservation methods and storage time points.

**FIG 2:**
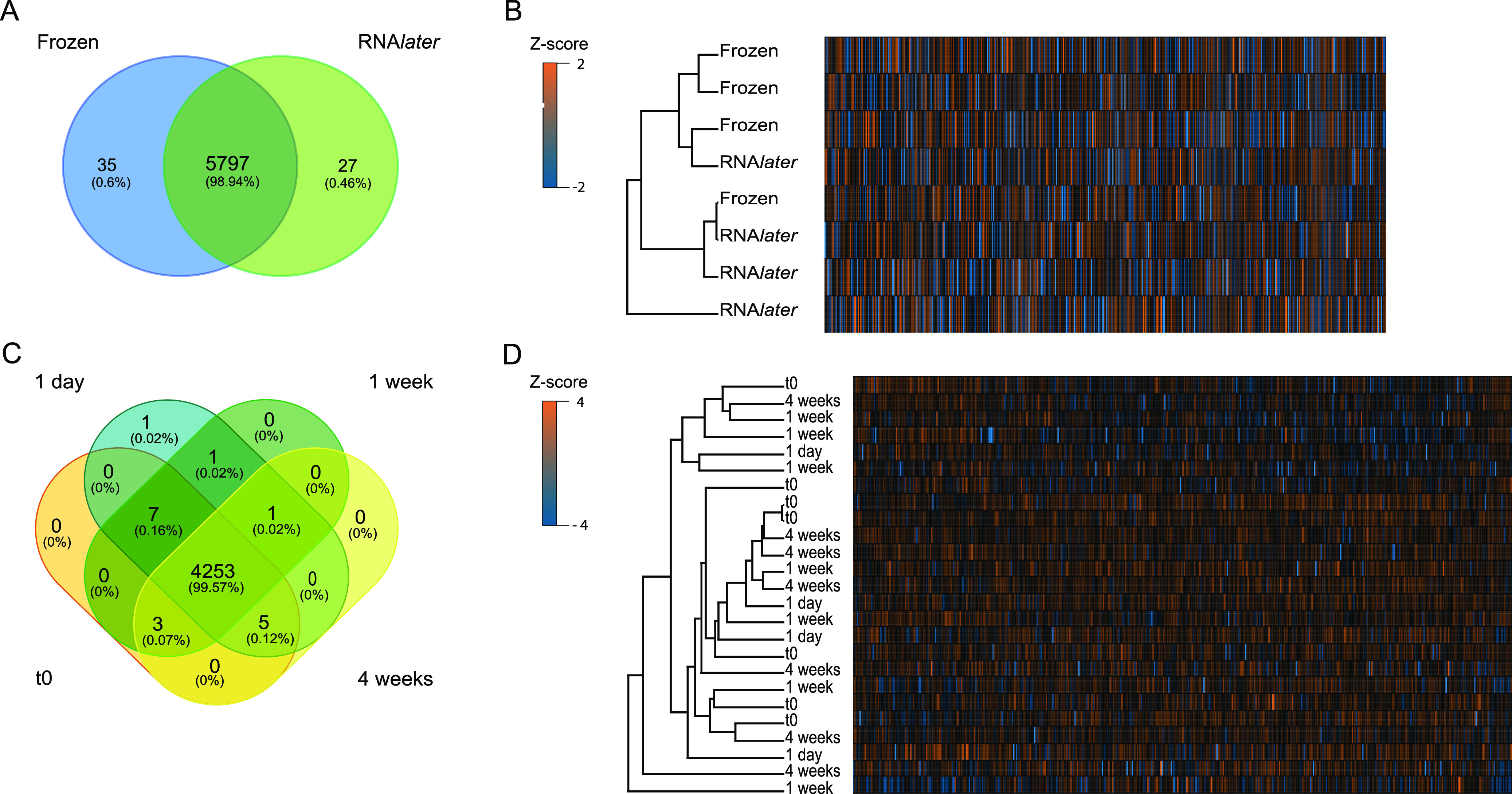
No systematic bias in protein identification and quantification between preservation methods and storage time points. (A and C) Unique and shared proteins identified in the preservation method comparison (A) and the RNA*later* time series (C). Only proteins that were retained after filtering for a 5% FDR and that were detected in at least 75% of samples in at least one group (preservation method/time point) were used for the analysis. (B and D) Hierarchical clustering of samples based on relative protein abundances for the preservation method comparison (B) and the RNA*later* time series (D). The same set of prefiltered proteins was used as in panels A and C and log_2_ transformed. The hierarchical clustering was based on Euclidean distance. Z-score values were calculated for each protein; positive Z-scores indicate a relative abundance higher than the mean, while negative Z-scores indicate a relative abundance lower than the mean.

Our results show that there is no systematic bias when sample preservation methods and time points are compared, based on overall protein identification and quantification. To identify systematic bias, we analyzed the data for proteins that were consistently present for only one of the preservation methods or time points, and we also applied hierarchical clustering based on protein abundances to check whether samples formed groups based on method or time point (Fig. 2A to D). For these analyses, we included only proteins that were consistently detected for at least one of the treatments/time points by filtering identified proteins at a false discovery rate (FDR) of 5%. Additionally, we required that proteins be detected in at least 75% of samples for at least one condition.

Of 9,326 proteins identified in the preservation method comparison (see Data File S3 in the supplemental material), 5,859 proteins remained after filtering and thus were considered to be consistently identified for at least one of the treatments. Of those 5,859 proteins, almost all (5,797 proteins) were shared between flash-frozen samples and samples preserved in RNA*later* (Fig. 2A). The hierarchical clustering of these samples based on protein abundances revealed multiple shared nodes in the dendrogram between flash-frozen and RNA*later*-preserved samples (Fig. 2B). In the case of a systematic bias introduced by the preservation method, we would expect separation of samples based on preservation method, with no shared nodes between preservation methods. These data suggest that the preservation methods did not introduce a systematic bias.

Of 7,036 proteins identified in the RNA*later* time series (see Data File S4), 4,271 were consistently identified and used for the overlap analysis. Of those 4,271 proteins, 4,253 proteins were shared across all four storage time points (Fig. 2C). We identified 1 unique protein for samples incubated for 1 day, whereas none of the other time points had unique proteins. Moreover, a few proteins were shared between two or three of the four different time points. The hierarchical clustering of these samples revealed multiple shared nodes between samples for all time points (1 day, 1 week, and 4 weeks) and samples for time zero (Fig. 2D). If a systematic bias had been introduced by long-term storage at room temperature, then we would expect separation of samples based on time point, with no shared nodes between test time points and time zero. These data suggest that long-term storage at room temperature did not introduce a systematic bias.

### No differences in relative abundances of individual proteins across preservation methods or time points.

**FIG 3:**
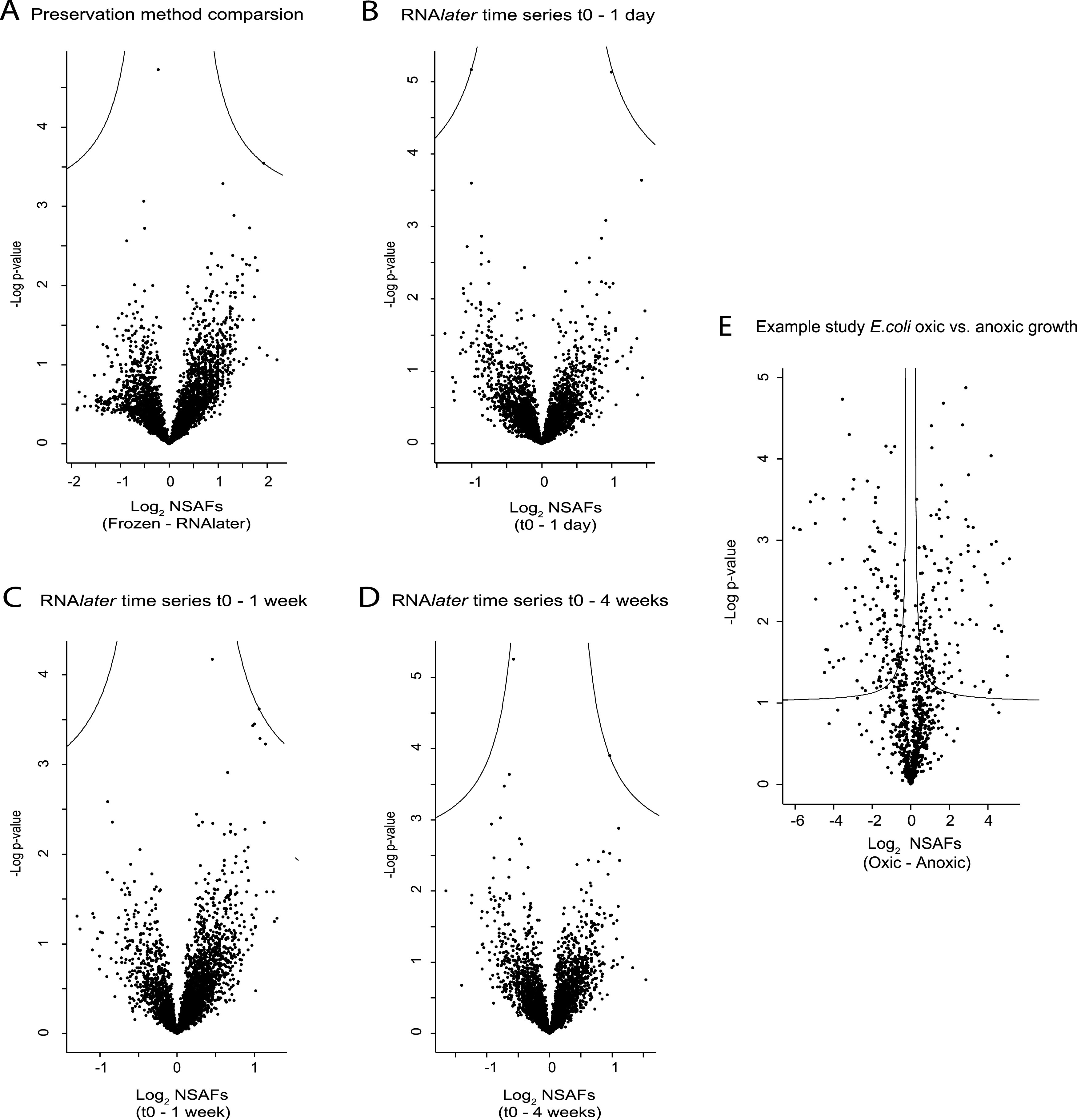
No differences in protein abundances between preservation methods and storage time points. Volcano plots of proteins filtered for a 5% FDR and at least 75% valid values in at least one group identified in the preservation method comparison (A), the RNA*later* time series (B to D), and an example study of E. coli grown under oxic and anoxic conditions (E). All proteins were plotted with log_2_(fold change) on the *x* axis and − log*P* on the *y* axis. The solid lines represent the significance threshold at 5% FDR and *S*_0_ of 0.1. Data points above the line represent proteins whose abundances significantly differed between comparisons, whereas data points below the line represent proteins whose abundances did not significantly differ between comparisons.

We evaluated relative protein abundances across preservation methods and storage time points to assess potential alterations in protein abundances introduced by method or time. For these analyses, we used the same data set as above, which included only proteins that were consistently detected for at least one of the treatments/time points. We used a two–sided Welch test to identify significant differences in protein abundances between methods and time points. Of the 5,859 consistently identified proteins in the preservation method comparison, no proteins appeared to be significantly different in abundance for any of the preservation methods (Fig. 3A). Of the 4,721 consistently identified proteins in the RNA*later* time series, no proteins differed significantly in abundance at any of the test time points, compared to time zero (Fig. 3B to D).

As an example to illustrate what results would look like if there were significant differences between treatments, we included an example study of Escherichia coli grown under either oxic or anoxic conditions (Fig. 3E). As expected, there were several differentially expressed proteins between the two growth conditions, as indicated by data points above the significance line in Fig. 3E. In summary, our analysis revealed no significant changes in protein abundances between the tested preservation methods, as well as between time zero and the storage time points of the RNA*later* time series.

### Effects on microbial community structure.

To investigate potential effects of preservation method or storage time on the representation of specific taxa in the metaproteome, we compared the proteinaceous biomass of each community member using a method adapted from the report by Kleiner et al. (43). This method enables calculations of proteinaceous biomass contributions of species in microbial communities by using protein abundances derived from metaproteomic analyses.

**FIG 4:**
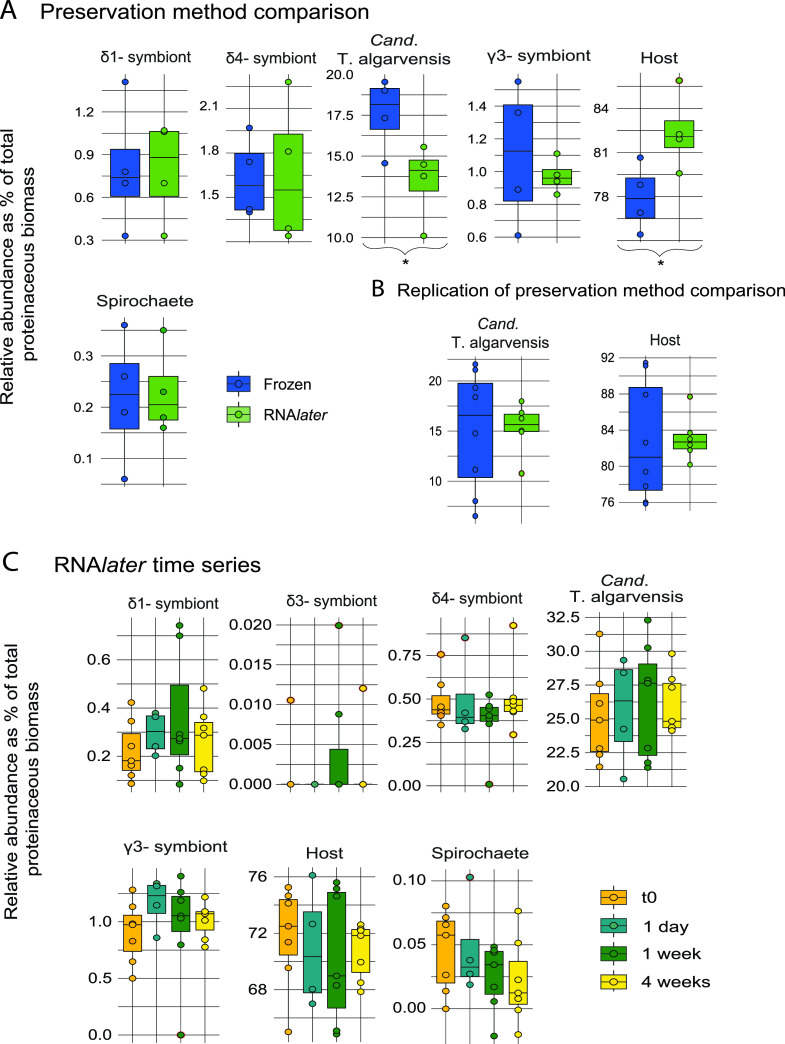
Per-species biomass estimates for members of the O. algarvensis symbiosis are mostly consistent for preservation methods and RNA*later* time series time points. Data for individual O. algarvensis specimens are shown for the preservation method comparison (A), a subset of the repetition of the preservation method comparison (B), and all storage time points of the RNA*later* time series (C). Taxa were quantified using the sum of PSM counts for each species. Identified proteins were filtered for a 5% FDR and at least 2 PUPs prior to counting as described previously (43). *, significant differences in per-species biomass (Student's *t* test, *P* < 0.05) (see Table S4 to S9 in the supplemental material).

**FIG 5:**
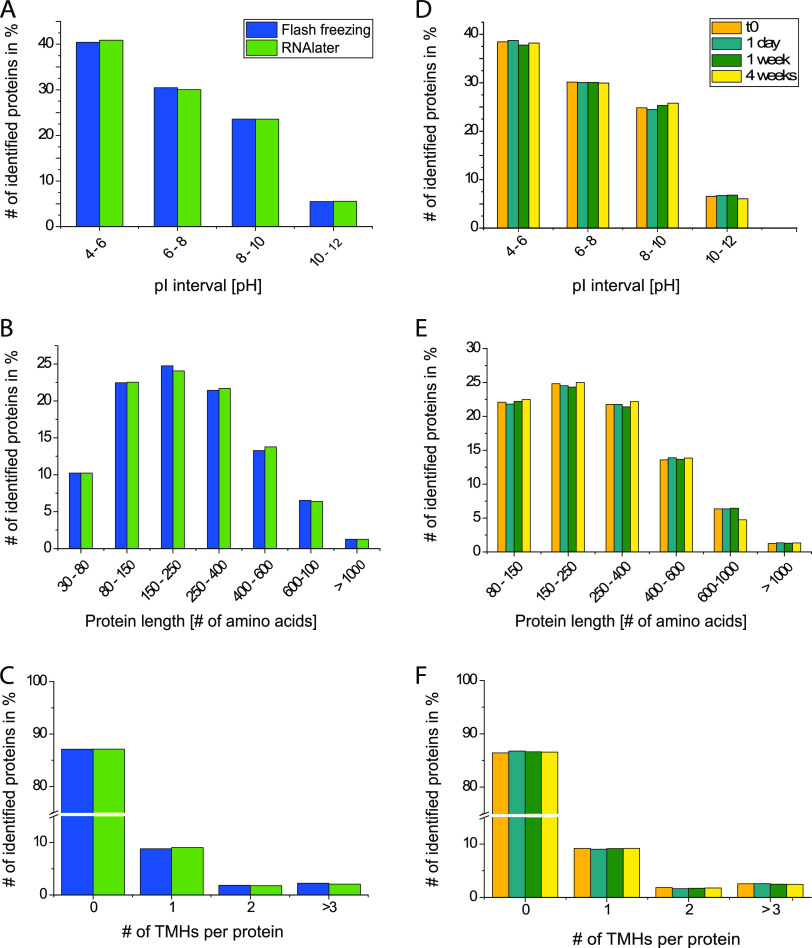
Preservation method and storage time in RNA*later* do not introduce any biases against proteins with specific biochemical properties. Identified proteins were filtered for a 5% FDR prior to counting. Only intervals contributing more than 1% of all identified proteins are displayed (e.g., pI 2 to 4 is not shown). (A and D) pI distribution of identified proteins for the preservation method comparison (A) and the RNA*later* time series (D). (B and E) Protein length distribution for the preservation method comparison (B) and the RNA*later* time series (E). (C and F) Number of predicted TMHs for the preservation method comparison (C) and the RNA*later* time series (F).

We found a small but significant difference in the proteinaceous biomass of the host and “*Cand*. T. algarvensis” in the preservation method comparison (Student's *t* test, *P* < 0.05) (Fig. 4A; also see Tables S4 and S5). The host’s biomass accounted for an average of 75.42% of total biomass in flash-frozen samples, while it was an average of 82.40% in RNA*later*-preserved samples. In contrast to the host’s biomass, the average biomass of “*Cand*. T. algarvensis” was higher in flash-frozen samples (17.61%) than in RNA*later*-preserved specimens (13.48%). None of the other symbionts showed a significant difference. To determine whether the preservation method truly affected the representation of specific taxa, we repeated this experiment with a fresh set of 16 O. algarvensis individuals to exclude biological variability between individual worms as a potential reason for the observed difference (Fig. 4B; also see Table S3 and Fig. S1). In this repeat experiment of the preservation method comparison, we did not observe any significant differences in proteinaceous biomass for the host or “*Cand*. T. algarvensis” or any other taxa (Fig. 5B; also see Tables S6 and S7 and Fig. S1). This finding suggests that there is either no effect or a small inconsistently occurring effect on taxon representation introduced by flash freezing and RNA*later* preservation.

In the RNA*later* time series, measured biomass abundances of species were relatively consistent between individual worms (Fig. 4C; also see Table S8). The only exception was the δ3 symbiont, which was detected in only 3 individuals; this finding is in line with the fact that this symbiont has been shown to be present in only a minority of individuals (8). None of the symbiont or host biomasses was significantly different when later time points were compared to time zero (Student's *t* test, *P* < 0.05) (Fig. 4C; also see Table S9), indicating that storage for up to 4 weeks in RNA*later* did not impact proteinaceous biomass measurements for individual species.

### No evidence for biases against proteins with specific biochemical properties.

The two tested preservation methods rely on distinct preservation mechanisms, which holds the potential for categorical loss or enrichment of proteins based on their biochemical properties. To evaluate the potential introduction of method-specific or storage-time-specific biases, we evaluated biochemical properties including protein size, isoelectric point (pI), and number of transmembrane helix (TMH) domains across all samples. In contrast to the overlap analysis shown in Fig. 2A and C, all proteins identified within an FDR of 5% were considered for this analysis.

We did not observe any significant differences in pI, protein size, or number of predicted TMHs for preservation methods (Fig. 5A to C) (Student's *t* test, *P* < 0.05) or storage time points (Fig. 5D to F) (Student's *t* test, *P* < 0.05). Counts within the respective intervals were almost identical for all examined parameters. For example, the average pI of proteins in flash-frozen samples was 6.86, while that in RNA*later*-preserved samples was 6.85, and the mean protein length was 296 amino acids for frozen samples and 297 amino acids for RNA*later*-preserved samples. Overall, our analysis showed that we recovered proteins with almost identical biochemical properties for both preservation methods and all storage time points. This suggests that RNA*later* robustly preserves proteins at room temperature for at least 4 weeks without introducing biases based on biochemical properties.

## DISCUSSION

We evaluated the protein preservation performance of flash freezing in liquid nitrogen and RNA*later* for metaproteomic analyses of field-collected samples. Our main finding was that the two preservation methods performed equally well and that storage time in RNA*later* for up to 4 weeks did not impact the quality of the metaproteomes. These findings are in line with the work of Saito et al. (32), who examined the performance of several preservation methods, including RNA*later*, to preserve cultures of the marine cyanobacterium *Synechococcus*, using flash-frozen samples as a control. Saito et al. (32) found that the number of identified proteins and relative protein abundances for RNA*later* were highly similar to those of flash-frozen controls, while the remaining preservatives showed significantly lower protein identification numbers. In addition, our findings provide validation for the successful application of RNA*later* in previous metaproteomic studies of bacterial-animal symbioses, such as those of the tube worm *Paraescarpia echinospica* (34), *Bathymodiolin* deep-sea mussels (36), the marine flatworm *Paracatenula* (35), and the marine ciliate *Kentrophoros* (37).

In contrast to the agreement with the work by Saito et al. (32), our work did not match with the results of Hickl et al. (33), who tested flash freezing and RNA*later* preservation using human fecal samples. Hickl et al. (33) observed differences in protein identifications and relative protein abundances, depending on the preservation method used. Based on the description of the methods used by Hickl et al. (33), however, it appears that the observed differences are due to inconsistent sample processing, rather than an actual performance difference for the tested preservation methods. The main inconsistency was that flash-frozen samples were homogenized and lysed by cryomilling, while RNA*later* samples were not subjected to any physical cell lysis. Use of physical lysis methods was shown previously to lead to major differences in recovery of DNA and proteins from members of microbial communities (44–46).

In addition to protein identification and quantification quality, there are other parameters that need to be taken into account when considering actual field deployment, some of which might vary depending on the sampling location and experimental design (Table 1). The comparison of flash freezing and preservation in RNA*later* showed that both methods stopped biological activity equally well. In addition, we found no alteration in preservation performance of RNA*later* over time and at room temperature. Flash freezing inhibits biological activity through a fast-freezing process at ultralow temperatures (−195.79°C), while RNA*later* contains high concentrations of ammonium sulfate, which causes protein precipitation and hence inactivation. Depending on the sample type and size, RNA*later* might take a few minutes to completely immerse the sample. While one could argue that this time holds the potential to introduce biases and protein degradation, our analyses showed that this concern is unsubstantiated. However, we recommend that these results be revalidated with larger animals or animals with an impermeable exoskeleton, because dissection into pieces smaller than the manufacturer’s recommendation of 0.5 cm may be required for fast and sufficient infusion of tissue.

**TABLE 1:** Comparison of suitability of flash freezing in liquid nitrogen and RNA*later* for protein preservation in field deployment

Requirement	Flash freezing	RNA*later*
Immediately stops biological activity	✓	✓
Preserves the whole metaproteome	✓	✓
Large temperature range	✗	✓
Wide range of sample types	✓	✓
Nonhazardous and easy to transport	✗	✓
Low cost	✗^*a*^	(✓)^*b*^

a The cost of dry ice or liquid N_2_ is relatively low but transport can be expensive, especially if flights are involved.

b RNA*later* can be substituted with RNA*later*-like solutions that can be produced in the laboratory at low cost.

Considering actual field deployment, an important parameter for a protein preservative is its ability to work in large temperature ranges, because weather conditions might vary significantly depending on the sampling location. We found that samples immersed in RNA*later* were robustly preserved for up to 4 weeks at room temperature (22°C to 23°C). Generally, when RNA*later* is used for RNA preservation, it has been shown to perform better at lower temperature (e.g., 4°C) (47). Therefore, we recommend storing samples as near this temperature as possible, and we consider 23°C the upper temperature limit for longer-term sample storage. If field conditions require longer-term storage above 23°C, then we recommend retesting of the impact of storage on metaproteome quality.

Another important aspect for field-compatible protein preservatives is that they should be nonhazardous and easy to transport to remote field locations. While this is the case for RNA*later*, which can be stored in plastic containers at room temperature, this does not hold true for liquid nitrogen, which can cause cryogenic burns, injury, or frostbite and may displace oxygen and cause rapid suffocation if handled improperly. As a consequence, liquid nitrogen shipment requires thorough planning prior to the sampling trip to ensure safe and smooth arrival at the destination. We were actually inspired to perform this study in part because, despite thorough planning, our liquid nitrogen dry shippers did not arrive at the field site several times in the past. Moreover, cryogenic shipment can easily exceed reasonable costs, since it requires a field-compatible cryogenic dry shipper and comes with high shipping costs, although the cost of liquid nitrogen itself is relatively low ($0.50 to 2.00 per liter). RNA*later*, on the other hand, is comparably expensive ($477 for 500 ml, according to the Thermo Fisher Scientific site on 21 February 2021); however, the amount of RNA*later* needed per sample is potentially very small, as the required RNA*later*/sample ratio is 10:1. This means, for example, that less than 100 μl of RNA*later* is needed per sample for the very small O. algarvensis worms. Additionally, the high cost of RNA*later* can be avoided by using a self-prepared RNA*later*-like solution that has been shown to perform as well as the commercially available solution (48).

Finally, once samples are brought back to the laboratory to await processing, they should be stored at 4°C in RNA*later* or, if the samples are targeted for long-term storage, the RNA*later* should be removed and samples should be frozen at −80°C. During removal of the RNA*later* prior to freezing of the samples, the samples should not be washed with a buffer, because the RNA*later* precipitation effect is reversible and proteases would become active again upon dilution of RNA*later*.

## MATERIALS AND METHODS

### Sampling and experimental setup.

We conducted two experiments to evaluate the performance of RNA*later* for protein preservation of field-collected samples, and we refer to them as preservation method comparison and RNA*later* time series. In addition, we fully repeated the preservation method comparison to confirm our results from the first experiment.

We collected and processed all samples as described previously (49). Briefly, samples were collected off the coast of Sant’ Andrea Bay (Elba, Italy) (42°48′26″N, 010°08′28″E) from shallow-water (6- to 8-m water depth) sediments next to seagrass beds. We collected 10 individuals of O. algarvensis in 2015 for the preservation method comparison, 16 individuals for the replication of the preservation study in August 2019, and 33 individuals for the RNA*later* time series in 2016 (see Tables S1 to S3 in the supplemental material). Live worms were transported in native Elba sediment and seawater to the Max Planck Institute for Marine Microbiology (Bremen, Germany), where we carefully removed the worms from the sediment and either froze the specimens at −80°C or immersed them in RNA*later* until further processing.

For the preservation method comparison, we flash froze 5 specimens of O. algarvensis in liquid nitrogen and then stored them at −80°C. We incubated the other 5 individuals in RNA*later* (Thermo Fisher Scientific). After 24 h, we removed the RNA*later* and stored the samples at −80°C until processing. We reproduced this setup in the replication of the preservation method comparison, with 9 flash-frozen and 7 RNA*later*-incubated O. algarvensis individuals.

For the RNA*later* time series, we incubated 33 O. algarvensis individuals in RNA*later*. Of those 33 individuals, 11 were incubated for 24 h in RNA*later* at 4°C (time zero), while 6, 8, and 8 individuals were incubated in RNA*later* at room temperature (22°C to 23°C) for an additional 24 h (time 1), 1 week (time 2), and 4 weeks (time 3) respectively. We removed RNA*later* after incubation and stored the samples at −80°C until further processing.

### Protein extraction, peptide preparation, and determination.

Samples for the preservation method comparison, replication of the preservation method comparison, and RNA*later* time series were processed slightly differently in terms of protein extraction. These steps are described separately for each study. Protein identification and quantification steps were identical in all studies and are thus described only once.

For the preservation method comparison, we prepared tryptic peptides following the filter-aided sample preparation (FASP) protocol, adapted from a previous report (50). We added 50 μl of SDT-lysis buffer (4% [wt/vol] SDS, 100 mM Tris-HCl [pH 7.6], 0.1 M dithiothreitol) to each sample and heated the samples to 90°C for 10 min. Samples were centrifuged for 5 min at 21,000 × *g*. We mixed 30 μl of each lysate with 200 μl UA solution (8 M urea in 0.1 M Tris/HCl [pH 8.5]) in a 10-kDa-molecular-weight-cutoff (MWCO) 500-μl centrifugal filter unit (VWR International) and centrifuged the mixture at 14,000 × *g* for 40 min. Next, we added 200 μl of UA solution and centrifuged the mixture again at 14,000 × *g* for 40 min. We added 100 μl of IAA solution (0.05 M iodoacetamide in UA solution) and then incubated the samples at 22°C for 20 min in the dark. We removed the IAA solution by centrifugation, followed by three wash steps with 100 μl of UA solution. Subsequently, we washed the filters three times with 100 μl of ABC buffer (50 mM ammonium bicarbonate). We added 1.6 μg of Pierce mass spectrometry (MS)-grade trypsin (Thermo Fisher Scientific) in 40 μl of ABC buffer to each filter. Filters were incubated overnight in a wet chamber at 37°C. The next day, we eluted the peptides by centrifugation at 14,000 × *g* for 20 min, followed by the addition of 50 μl of 0.5 M NaCl and another centrifugation step. Peptides were quantified using the Pierce micro-bicinchoninic acid (BCA) kit (Thermo Fisher Scientific), following the instructions of the manufacturer.

We processed samples for the preservation method replication and the RNA*later* time series similarly to samples for the preservation method comparison, with the following modifications: we added 60 μl of SDT-lysis buffer instead of 50 μl and boiled the samples at 95°C for 10 min. To minimize sample loss, we did not perform the 5-min centrifugation at 21,000 × *g* described in the original protocol (50) and instead mixed the complete 60 μl of each lysate with 400 μl of UA solution in a 10-kDa-MWCO 500-μl centrifugal filter unit. All subsequent steps were identical to the sample preparation for the preservation method comparison with the exception that we added 0.62 μg and 0.54 μg of Pierce MS-grade trypsin (Thermo Fisher Scientific) in 40 μl of ABC buffer to each filter for the repetition of the preservation method comparison and the RNA*later* time series, respectively.

### One-dimensional liquid chromatography–tandem MS.

All samples were analyzed by one-dimensional liquid chromatography-tandem MS (1D-LC-MS/MS). Detailed instrument setups, gradients, and methods are specified in Data File S1 in the supplemental material. In brief, all samples were loaded onto a C_18_ Acclaim PepMap 100 precolumn and separated on an Easy-Spray PepMap C_18_ analytical column (75 μm by 75 cm; Thermo Fisher Scientific) using reverse-phase LC. Eluting peptides were ionized with electrospray ionization, and mass spectra were acquired using a data-dependent acquisition method in a Q-Exactive Orbitrap mass spectrometer (Thermo Fisher Scientific).

### Protein identification and quantification.

We downloaded an existing custom protein sequence database for the O. algarvensis symbiosis (49) from the PRIDE repository (PRIDE accession number PXD007510) and used it for protein identification. The database contained 1,439,794 protein sequences, including host and symbiont proteins, as well as a cRAP protein sequence database (http://www.thegpm.org/crap) of common laboratory contaminants. We performed searches of the MS/MS spectra against this database with the Sequest HT node in Proteome Discoverer version 2.2.0.388 (Thermo Fisher Scientific), as described by Gruber-Vodicka et al. (51). The following parameters were used: trypsin (full), maximum of 2 missed cleavages, 10 ppm precursor mass tolerance, 0.1-Da fragment mass tolerance, and maximum of 3 equal dynamic modifications per peptide, namely, oxidation on M (+15.995 Da), carbamidomethyl on C (+57.021 Da), and acetyl on the protein N terminus (+42.011 Da). FDRs for peptide spectral matches (PSMs) were calculated and filtered using the Percolator node in Proteome Discoverer (52). Percolator was run with a maximum Delta CN of 0.05, a strict target FDR of 0.01, a relaxed target FDR of 0.05, and validation based on *q* value. The Protein FDR Validator node in Proteome Discoverer was used to calculate *q* values for inferred proteins based on the results from a search against a target-decoy database. Proteins with *q* values of <0.01 were categorized as high-confidence identifications, and proteins with *q* values of 0.01to 0.05 were categorized as medium-confidence identifications. We combined search results for all samples into a multiconsensus report in Proteome Discoverer, and only proteins identified with medium or high confidence were retained, resulting in an overall protein-level FDR of 5%. For protein quantification, normalized spectral abundance factors (NSAFs) (53) were calculated for each species and multiplied by 100, to give the relative protein abundance as a percentage.

### Outlier identification and removal.

We classified samples as outliers if at least two of the following criteria were met: (i) the total ion chromatogram intensity was below 1 × 10^9^; (ii) the proportional number of standard deviations (SDs) above and below the mean (Z-score) of the number of identified proteins (filtered for a 5% FDR) was greater than ±1; or (iii) the number of identified proteins (filtered for a 5% FDR) was more than 1 SD below the mean number of identified proteins of all samples within a group (see Data File S2). In addition, we applied the generalized extreme studentized deviate (ESD) test (significance level of 0.5, with a maximum of 10 outliers) to the number of identified proteins in the RNA*later* time series for outlier identification. This procedure was not applied to the preservation method comparison and the replication of the preservation method comparison due to an insufficient number of replicates. In total, we identified 2 samples of the preservation method comparison, 2 samples of the repetition of the method comparisons, and 8 samples of the RNA*later* time series as outliers (see Data File S2). Identified outliers were excluded from all subsequent analyses. The identification of these outliers was expected, because differences in biomass and age of the processed specimens, as well as slight variations in sample handling, can lead to lower protein recovery during the extraction protocol.

In addition, we checked the metaproteomes for evidence of accidental sampling of the cooccurring marine gutless oligochaete Olavius ilvae (54). O. ilvae cannot be easily distinguished from O. algarvensis during sampling because O. algarvensis and O. ilvae are highly similar in size, shape, and color. However, they harbor distinct symbionts, which can be used to distinguish between the species (55). To test whether any of our samples was a specimen of O. ilvae, we created a custom database including protein sequences of the α7 symbiont, “*Candidatus* Thiosymbion” sp., γ3 symbiont, and δ3 symbiont of O. ilvae. In addition, we included protein sequences of “*Cand*. T. algarvensis” and the δ1 symbiont of O. algarvensis for testing. The database was then loaded into Proteome Discoverer, and proteins were identified as described above. One sample of the RNA*later* time series was identified as O. ilvae and thus removed as an outlier (see Data File S2).

### Data analysis.

To determine which identified proteins were shared by all samples or unique to specific treatments/time points, we loaded the 5% FDR-filtered PSM multiconsensus files into Perseus 1.6.5.0 (56), filtered out proteins that did not have at least 75% valid values (greater than 0) in at least one group, and log_2_ transformed the data. We then calculated the overlap protein sets with the numerical Venn function in Perseus and visualized the results with a Venn calculation tool from Ghent University (http://bioinformatics.psb.ugent.be/webtools/Venn) using the default settings.

For hierarchical clustering, we loaded the 5% FDR-filtered NSAF multiconsensus files into Perseus, filtered out proteins that did not have at least 75% valid values in at least one group, and log_2_ transformed the data. We replaced invalid values with a constant value and Z-score normalized the resulting matrix by rows (proteins). Subsequently, we performed hierarchical clustering with the following settings: Euclidean distance, preprocessing with k-means, and average linkage.

For the analysis of differential protein abundances, we loaded the 5% FDR-filtered PSM multiconsensus files into Perseus and filtered for 75% valid values in at least one group, to use only consistently identified proteins. We then added 1 to every PSM value before performing the NSAF calculation, to protect against issues with missing values. We grouped samples by preservation method/time point and performed a two-sided Welch’s *t* test using a permutation-based FDR of 5% to account for multiple hypothesis testing. We log_2_ transformed the data and used the resulting matrix as input data for volcano plots based on a *t* test with an FDR of 0.05 and an *S*_0_ value of 0.1. *S*_0_ is a constant that modifies the FDR based significance threshold to consider the size of the fold change.

We calculated relative abundances for each species in the symbiosis using the method for assessing the proteinaceous biomass described by Kleiner et al. (43), with the following modification. Instead of using FidoCT for protein inference in Proteome Discoverer and filtering for proteins with at least 2 protein unique peptides (PUPs), we used Sequest HT for protein inference and filtered for proteins with at least 2 PUPs. We visualized the results with the ggplot package in R (57, 58).

To assess the biochemical properties of all identified proteins, we obtained the number of amino acids and predicted pI values from Proteome Discoverer. We predicted TMHs with the TMHMM Server v2.0 (59). Protein sequences of all identified proteins for each study were used as input data.

### Data availability.

The metaproteomics MS data and protein sequence database have been deposited in the ProteomeXchange Consortium via the PRIDE partner repository (60), with the following data set identifiers: preservation method comparison, PXD014591; replication of the method preservation method comparison, PXD026631; RNA*later* time series, PXD014881; E. coli grown under oxic and anoxic conditions, PXD024288.
