# Supplementary material for: Evaluation of RNAlater as a Field-Compatible Preservation Method for Metaproteomic Analyses of Bacterium-Animal Symbioses
Source: Microbiol Spectr. 2021 Oct 27;9(2):e01429-21. doi: 10.1128/Spectrum.01429-21 (PMC8549751; doi:10.1128/Spectrum.01429-21)
Supplement: SUPPLEMENTAL FILE 1 — Supplemental material. Download Spectrum.01429-21-s0001.pdf, PDF file, 0.4 MB [file spectrum.01429-21-s0001.pdf]

Supplemental material for "Evaluation of RNA*later*™ as a field-compatible preservation method for metaproteomic analyses of bacteria-animal symbioses"

Authors: Marlene Jensen[1], Juliane Wippler[2], Manuel Kleiner[1]

Affiliations:

1: North Carolina State University; Department of Plant & Microbial Biology, 112 Derieux Pl, 4510 Thomas Hall, Raleigh, NC 27695

2: Symbiosis Department, Max Planck Institute for Marine Microbiology, Celsiusstr. 1, D-28359, Bremen, Germany

#corresponding authors
manuel_kleiner(at)ncsu(dot)edu, mjensen2(at)ncsu(dot)edu

Table S1: Sample overview for the preservation method comparison

Sample ID	Sample storage	Species
1	Flash freeing in liquid nitrogen and stored @ -80°C	*O. algarvensis*
2	Flash freeing in liquid nitrogen and stored @ -80°C	*O. algarvensis*
3	Flash freeing in liquid nitrogen and stored @ -80°C	*O. algarvensis*
4	Flash freeing in liquid nitrogen and stored @ -80°C	*O. algarvensis*
5	Flash freeing in liquid nitrogen and stored @ -80°C	*O. algarvensis*
11	RNA*later*™ incubated for 24 hours, and stored @ -80 °C	*O. algarvensis*
12	RNA*later*™ incubated for 24 hours and stored @ -80 °C	*O. algarvensis*
13	RNA*later*™ incubated for 24 hours and stored @ -80 °C	*O. algarvensis*
14	RNA*later*™ incubated for 24 hours and stored @ -80 °C	*O. algarvensis*
15	RNA*later*™ incubated for 24 hours and stored @ -80 °C	*O. algarvensis*

Table S2: Sample overview for the replication of the preservation method comparison

Sample ID	Sample storage	Species
1_F	Flash freeing in liquid nitrogen and stored @ -80°C	*O. algarvensis*
2_F	Flash freeing in liquid nitrogen and stored @ -80°C	*O. algarvensis*
3_F	Flash freeing in liquid nitrogen and stored @ -80°C	*O. algarvensis*
4_F	Flash freeing in liquid nitrogen and stored @ -80°C	*O. algarvensis*
9_F	Flash freeing in liquid nitrogen and stored @ -80°C	*O. algarvensis*
10_F	Flash freeing in liquid nitrogen and stored @ -80°C	*O. algarvensis*
11_F	Flash freeing in liquid nitrogen and stored @ -80°C	*O. algarvensis*
12_F	Flash freeing in liquid nitrogen and stored @ -80°C	*O. algarvensis*
13_F	Flash freeing in liquid nitrogen and stored @ -80°C	*O. algarvensis*
14_F	Flash freeing in liquid nitrogen and stored @ -80°C	*O. algarvensis*
t1_21_R	RNA*later*™ incubated for 24 hours and stored @ -80 °C	*O. algarvensis*
t1_22_R	RNA*later*™ incubated for 24 hours and stored @ -80 °C	*O. algarvensis*
t1_23_R	RNA*later*™ incubated for 24 hours and stored @ -80 °C	*O. algarvensis*
t1_24_R	RNA*later*™ incubated for 24 hours and stored @ -80 °C	*O. algarvensis*
t1_25_R	RNA*later*™ incubated for 24 hours and stored @ -80 °C	*O. algarvensis*
t1_26_R	RNA*later*™ incubated for 24 hours and stored @ -80 °C	*O. algarvensis*
t1_27_R	RNA*later*™ incubated for 24 hours and stored @ -80 °C	*O. algarvensis*
t1_28_R	RNA*later*™ incubated for 24 hours and stored @ -80 °C	*O. algarvensis*
t1_29_R	RNA*later*™ incubated for 24 hours and stored @ -80 °C	*O. algarvensis*
t1_30_R	RNAlater™ incubated for 24 hours and stored @ -80 °C	*O. algarvensis*

Table S3: Sample overview for the RNA*later*™ time series

Sample ID	Time point	Sample storage	Species
2	t0	Fixation in RNA*later*™ for 24 h at 4 °C, then transfer to -80 °C	*O. algarvensis*
3	t0	Fixation in RNA*later*™ for 24 h at 4 °C, then transfer to -80 °C	*O. algarvensis*
5	t0	Fixation in RNA*later*™ for 24 h at 4 °C, then transfer to -80 °C	*O. algarvensis*
7	t0	Fixation in RNA*later*™ for 24 h at 4 °C, then transfer to -80 °C	*O. algarvensis*
8	t0	Fixation in RNA*later*™ for 24 h at 4 °C, then transfer to -80 °C	*O. algarvensis*
13	t0	Fixation in RNA*later*™ for 24 h at 4 °C, then transfer to -80 °C	*O. algarvensis*
14	t0	Fixation in RNA*later*™ for 24 h at 4 °C, then transfer to -80 °C	*O. algarvensis*
15	t0	Fixation in RNA*later*™ for 24 h at 4 °C, then transfer to -80 °C	*O. algarvensis*
16	t0	Fixation in RNA*later*™ for 24 h at 4 °C, then transfer to -80 °C	*O. algarvensis*
17	t0	Fixation in RNA*later*™ for 24 h at 4 °C, then transfer to -80 °C	*O. algarvensis*
18	t0	Fixation in RNA*later*™ for 24 h at 4 °C, then transfer to -80 °C	*O. ilvae*
46	t1	Kept in RNA*later*™ at RT for 24 hours, then transfer to -80 °C	*O. algarvensis*
47	t1	Kept in RNA*later*™ at RT for 24 hours, then transfer to -80 °C	*O. algarvensis*
48	t1	Kept in RNA*later*™ at RT for 24 hours, then transfer to -80 °C	*O. algarvensis*
49	t1	Kept in RNA*later*™ at RT for 24 hours, then transfer to -80 °C	*O. algarvensis*

51	t1	Kept in RNA*later*™ at RT for 24 hours, then transfer to -80 °C	*O. algarvensis*
52	t1	Kept in RNA*later*™ at RT for 24 hours, then transfer to -80 °C	*O. algarvensis*
32	t2	Kept in RNA*later*™ at RT for 7 days, then transfer to -80 °C	*O. algarvensis*
34	t2	Kept in RNA*later*™ at RT for 7 days, then transfer to -80 °C	*O. algarvensis*
36	t2	Kept in RNA*later*™ at RT for 7 days, then transfer to -80 °C	*O. algarvensis*
38	t2	Kept in RNA*later*™ at RT for 7 days, then transfer to -80 °C	*O. algarvensis*
40	t2	Kept in RNA*later*™ at RT for 7 days, then transfer to -80 °C	*O. algarvensis*
41	t2	Kept in RNA*later*™ at RT for 7 days, then transfer to -80 °C	*O. algarvensis*
42	t2	Kept in RNA*later*™ at RT for 7 days, then transfer to -80 °C	*O. algarvensis*
44	t2	Kept in RNA*later*™ at RT for 7 days, then transfer to -80 °C	*O. algarvensis*
19	t3	Kept in RNA*later*™ at RT for 4 weeks, then transfer to -80 °C	*O. algarvensis*
21	t3	Kept in RNA*later*™ at RT for 4 weeks, then transfer to -80 °C	*O. algarvensis*
23	t3	Kept in RNA*late*r™ at RT for 4 weeks, then transfer to -80 °C	*O. algarvensis*
24	t3	Kept in RNA*later*™ at RT for 4 weeks, then transfer to -80 °C	*O. algarvensis*
25	t3	Kept in RNA*later*™ at RT for 4 weeks, then transfer to -80 °C	*O. algarvensis*
26	t3	Kept in RNA*later*™ at RT for 4 weeks, then transfer to -80 °C	*O. algarvensis*
27	t3	Kept in RNA*late*r™ at RT for 4 weeks, then transfer to -80 °C	*O. algarvensis*
30	t3	Kept in RNA*later*™ at RT for 4 weeks, then transfer to -80 °C	*O. algarvensis*

Table S4: Metaproteomics based measurements of per-species biomass of members of the *O. algarvensis* symbiosis. Data are shown for *O. algarvensis* specimens used in the preservation method comparison. Calculations are based on a modified method of Kleiner et al. (Kleiner et al. 2017). Taxa were quantified using the sum of PSM counts for each species. Identified proteins were filtered for 5% FDR and at least 2 protein unique peptides (PUP) prior to counting. "Unassigned symbionts" is the total abundance of proteins identified with protein sequences that could not be assigned to a specific symbiont in previous metagenomics studies.

Organism	Biomass frozen samples [%], n= 4	Biomass RNA*later*™ preserved samples [%], n = 4
δ1-symbiont	0.78	0.70
δ1-symbiont	1.41	1.06
δ1-symbiont	0.70	0.33
δ1-symbiont	0.33	1.07
δ3-symbiont	0.00	0.00
δ3-symbiont	0.00	0.00
δ3-symbiont	0.00	0.00
δ3-symbiont	0.00	0.00
δ4-symbiont	1.74	1.29
δ4-symbiont	1.97	1.81
δ4-symbiont	1.40	2.28
δ4-symbiont	1.42	1.24
Cand. T. algarvensis	19.54	14.48
Cand. T. algarvensis	14.56	15.56
Cand. T. algarvensis	19.01	13.77

Cand. T. algarvensis	17.33	10.11
γ3-symbiont	1.36	0.86
γ3-symbiont	0.61	1.11
γ3-symbiont	0.89	0.94
γ3-symbiont	1.55	0.98
Host	75.42	81.91
Host	80.65	79.58
Host	76.91	82.24
Host	78.80	85.89
Spirochaete	0.36	0.18
Spirochaete	0.19	0.23
Spirochaete	0.26	0.16
Spirochaete	0.06	0.35
Unassigned symbionts	0.79	0.59
Unassigned symbionts	0.63	0.65
Unassigned symbionts	0.83	0.28
Unassigned symbionts	0.51	0.36

Table S5: Two-tailed Student's t-test on the calculated per-species biomass of members of the *O. algarvensis* symbiosis. Data are shown for *O. algarvensis* specimens used in the preservation method comparison.

Organism	p-value Frozen vs. RNA*later* ™
δ1-symbiont	0.97
δ4-symbiont	0.95
*Cand.*T.algarvensis	0.04
γ3-symbiont	0.60
Host	0.04
Spirochaete	0.85
Unassigned symbionts	0.11

Table S6: Metaproteomics based measurements of per-species biomass of members of the *O. algarvensis* symbiosis. Data are shown for *O. algarvensis* specimens used in the replication of the preservation method comparison. Calculations are based on a modified method of Kleiner et al. (Kleiner et al. 2017). Taxa were quantified using the sum of PSM counts for each species. Identified proteins were filtered for 5% FDR and at least 2 PUP prior to counting. "Unassigned symbionts" is the total abundance of proteins identified with protein sequences that could not be assigned to a specific symbiont in previous metagenomics studies.

Organism	Biomass frozen samples [%] n= 8	Biomass RNA*later*™ preserved samples [%] n = 6
δ1-symbiont	0.09	0.25
δ1-symbiont	0.51	0.10
δ1-symbiont	0.25	0.13
δ1-symbiont	0.08	0.02

δ1-symbiont	0.06	0.16
δ1-symbiont	0.06	0.10
δ1-symbiont	0.54	NA
δ1-symbiont	0.21	NA
δ4-symbiont	1.14	0.39
δ4-symbiont	0.00	0.57
δ4-symbiont	1.00	0.40
δ4-symbiont	0.15	0.50
δ4-symbiont	0.23	0.31
δ4-symbiont	0.78	0.36
δ4-symbiont	1.02	NA
δ4-symbiont	0.84	NA
Cand. T. algarvensis	14.77	10.77
Cand. T. algarvensis	6.52	17.97
Cand. T. algarvensis	21.11	15.04
Cand. T. algarvensis	8.01	16.82
Cand. T. algarvensis	11.15	14.93
Cand. T. algarvensis	18.38	16.26
Cand. T. algarvensis	19.32	NA
Cand. T. algarvensis	21.66	NA
γ3-symbiont	0.77	0.57
γ3-symbiont	0.56	0.57
γ3-symbiont	0.68	0.74
γ3-symbiont	0.31	0.50
γ3-symbiont	0.43	0.69
γ3-symbiont	0.65	0.57
γ3-symbiont	0.94	NA
γ3-symbiont	0.46	NA
Host	82.62	87.70
Host	91.46	80.18
Host	76.01	83.01
Host	91.13	81.78
Host	87.93	83.70
Host	79.38	82.36
Host	77.79	NA
Host	75.85	NA
Spiro	0.31	0.05
Spiro	0.00	0.10
Spiro	0.17	0.10
Spiro	0.08	0.04
Spiro	0.06	0.03
Spiro	0.07	0.12
Spiro	0.07	NA
Spiro	0.17	NA

Unassigned	0.30	0.26
Unassigned	0.95	0.51
Unassigned	0.77	0.58
Unassigned	0.23	0.34
Unassigned	0.15	0.17
Unassigned	0.69	0.25
Unassigned	0.31	NA
Unassigned	0.81	NA

Table S7: Two-tailed Student's t-test on the calculated per-species biomass of members of the *O. algarvensis* symbiosis. Data are shown for *O. algarvensis* specimens used in the replication of the preservation method comparison

Organism	p-value<br>Frozen vs. RNA*later* ™
δ1-symbiont	0.23
δ4-symbiont	0.21
*Cand.*T.algarvensis	0.94
γ3-symbiont	0.95
Host	0.89
Spirochaete	0.36
Unassigned symbionts	0.20

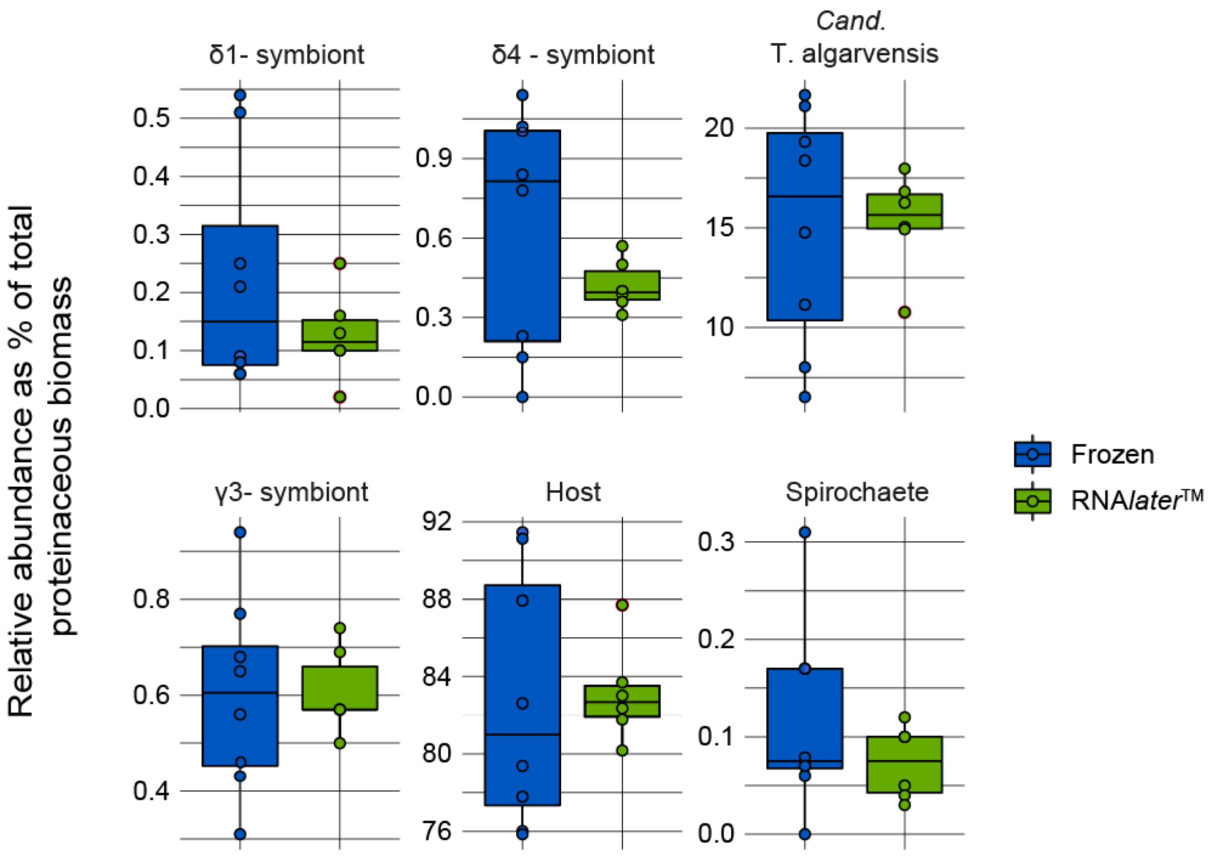

Figure S1: Metaproteomics based measurements of per-species biomass of members of the *O. algarvensis* symbiosis. Data for individual *O. algarvensis* specimens are shown for specimens used in the preservation method comparison**.** Taxa were quantified using the sum of PSM counts for each species respectively. Identified proteins were filtered for 5% FDR and at least 2 PUP prior to counting.

Table S8: Metaproteomics based measurements of per-species biomass of members of the *O. algarvensis* symbiosis. Data are shown for *O. algarvensis* specimens used in the RNA*later*™ time series**.** Calculations are based on a modified method of Kleiner et al. (Kleiner et al. 2017). Taxa were quantified using the sum of PSM counts for each species respectively. Identified proteins were filtered for 5% FDR and at least 2 PUP prior to counting. "Unassigned symbionts" is the total abundance of proteins identified with protein sequences that could not be assigned to a specific symbiont in previous metagenomics studies.

Organism	Biomass t0 [%] n= 7	Biomass 1 day [%] n= 4	Biomass 1 week [%] n= 7	Biomass 4 weeks [%] n= 7
δ1-symbiont	0.12	0.24	0.08	0.29
δ1-symbiont	0.16	0.20	0.74	0.32
δ1-symbiont	0.24	0.38	0.26	0.14
δ1-symbiont	0.42	0.36	0.15	0.10
δ1-symbiont	0.09	NA	0.29	0.48
δ1-symbiont	0.18	NA	0.70	0.13
δ1-symbiont	0.35	NA	0.27	0.36
δ3-symbiont	0.00	0.00	0.00	0.00
δ3-symbiont	0.00	0.00	0.00	0.00
δ3-symbiont	0.00	0.00	0.00	0.00

δ3-symbiont	0.00	0.00	0.00	0.00
δ3-symbiont	0.00	NA	0.01	0.01
δ3-symbiont	0.01	NA	0.00	0.00
δ3-symbiont	0.00	NA	0.02	0.00
δ4-symbiont	0.76	0.85	0.39	0.46
δ4-symbiont	0.44	0.33	0.01	0.43
δ4-symbiont	0.42	0.42	0.52	0.51
δ4-symbiont	0.58	0.37	0.36	0.29
δ4-symbiont	0.40	NA	0.40	0.43
δ4-symbiont	0.46	NA	0.45	0.92
δ4-symbiont	0.35	NA	0.46	0.49
*Cand.*T. algarvensis	31.28	20.55	22.84	27.92
*Cand.*T. algarvensis	27.58	29.34	32.29	24.31
*Cand.*T. algarvensis	21.46	28.41	21.40	24.39
*Cand.*T. algarvensis	22.81	24.25	27.86	29.82
*Cand.*T. algarvensis	24.91	NA	21.76	27.33
*Cand.*T. algarvensis	26.14	NA	30.26	24.14
*Cand.*T. algarvensis	22.36	NA	27.64	24.82
γ3-symbiont	0.83	0.86	1.05	1.22
γ3-symbiont	0.98	1.34	0.00	1.09
γ3-symbiont	1.28	1.32	0.80	0.84
γ3-symbiont	0.65	1.15	1.26	1.07
γ3-symbiont	0.50	NA	1.19	1.09
γ3-symbiont	1.13	NA	1.40	0.78
γ3-symbiont	0.97	NA	1.03	1.01
Host	65.01	76.11	74.63	69.95
Host	69.55	67.01	64.85	72.61
Host	75.25	68.04	75.60	71.83
Host	74.12	72.65	68.31	68.50
Host	72.50	NA	75.15	72.32
Host	71.37	NA	65.09	72.20
Host	74.65	NA	68.98	67.86
Spirochaete	0.11	0.04	0.07	0.03
Spirochaete	0.02	0.05	0.07	0.08
Spirochaete	0.08	0.13	0.04	0.10
Spirochaete	0.04	0.06	0.03	0.04
Spirochaete	0.05	NA	0.06	0.00
Spirochaete	0.09	NA	0.07	0.05
Spirochaete	0.10	NA	0.00	0.02
Unassigned symbionts	1.90	1.35	0.94	0.70
Unassigned symbionts	1.26	1.74	2.04	1.38
Unassigned symbionts	1.26	1.31	1.38	1.46
Unassigned symbionts	1.38	1.16	2.04	1.57
Unassigned symbionts	1.55	NA	1.13	1.47
Unassigned symbionts	0.62	NA	2.03	1.82

Unassigned symbionts	1.22	NA	1.60	0.82

Table S9: Two-tailed Student's t-test on the calculated per-species biomass of members of the *O. algarvensis* symbiosis. Data are shown for *O. algarvensis* specimens used in the RNA*later*™ time series.

Organism	p-value t0 vs. 1 day	p-value t0 vs. 1 week	p-value t0 vs. 4 weeks
δ1-symbiont	0.28	0.25	0.61
δ3-symbiont	0.36	0.45	0.93
δ4-symbiont	0.97	0.18	0.85
Cand. T. algarvensis	0.87	0.62	0.58
γ3-symbiont	0.13	0.79	0.39
Host	0.75	0.54	0.52
Spirochaete	0.94	0.21	0.19
Unassigned symbionts	0.70	0.24	0.98
